# Baroreflex modulation of muscle sympathetic nerve activity at rest does not differ between morning and afternoon

**DOI:** 10.3389/fnins.2015.00312

**Published:** 2015-09-02

**Authors:** Sarah L. Hissen, Vaughan G. Macefield, Rachael Brown, Trevor Witter, Chloe E. Taylor

**Affiliations:** ^1^School of Science and Health, University of Western SydneySydney, NSW, Australia; ^2^School of Medicine, University of Western SydneySydney, NSW, Australia; ^3^Neuroscience Research AustraliaSydney, NSW, Australia; ^4^Centre for Translational Physiology, University of OtagoWellington, New Zealand

**Keywords:** muscle sympathetic nerve activity, blood pressure, baroreflex sensitivity, diurnal variation, circadian

## Abstract

The incidence of cardiovascular events is significantly higher in the morning than other times of day. This has previously been associated with poor blood pressure control via the cardiac baroreflex. However, it is not known whether diurnal variation exists in vascular sympathetic baroreflex function, in which blood pressure is regulated via muscle sympathetic nerve activity (MSNA). The aim of this study was to compare vascular sympathetic baroreflex sensitivity (BRS) in the same participants between the morning and afternoon. In 10 participants (mean age 22 ± 2.9 years), continuous measurements of blood pressure, heart rate and MSNA were made during 10 min of rest in the morning (between 0900 and 1000 h) and afternoon (between 1400 and 1500 h). Spontaneous vascular sympathetic BRS was quantified by plotting MSNA burst incidence against diastolic pressure (vascular sympathetic BRS_inc_), and by plotting total MSNA against diastolic pressure (vascular sympathetic BRS_total_). Significant vascular sympathetic BRS_inc_ and vascular sympathetic BRS_total_ slopes were obtained for 10 participants at both times of day. There was no significant difference in vascular sympathetic BRS_inc_ between morning (−2.2 ± 0.6% bursts/mmHg) and afternoon (−2.5 ± 0.2% bursts/mmHg; *P* = 0.68) sessions. Similarly, vascular sympathetic BRS_total_ did not differ significantly between the morning (−3.0±0.5 AU/beat/mmHg) and afternoon (−2.9 ± 0.4 AU/beat/mmHg; *P* = 0.89). It is concluded that in healthy, young individuals baroreflex modulation of MSNA at rest does not differ between the morning and afternoon. The results indicate that recording MSNA at different times of the day is a valid means of assessing sympathetic function.

## Introduction

The incidence of cardiovascular and cerebrovascular events is higher in the morning than at any other time of day (Elliott, 1998; Muller et al., 1989). The morning is associated with a surge in blood pressure alongside elevated heart rate, blood viscosity and platelet aggregability, which are thought to increase the risk of transient ischaemic events (Muller et al., 1989). Acute increases in blood pressure can cause rupture of atherosclerotic plaques from the arterial wall and arterial thrombosis, leading to myocardial infarction and stroke. Poor blood pressure control in the morning may therefore play a role in the elevated risk of such events.

The baroreflex provides the principle means of buffering acute changes in blood pressure. It operates as a negative feedback loop responding to the activation of stretch sensitive receptors in the carotid sinus and aortic arch (baroreceptors), which project to the nucleus tractus solitarious (NTS) in the medulla via the glossopharyngeal and vagus nerves (Andresen and Kunze, 1994). The baroreflex can be described as having two distinct arms; the cardiac and vascular sympathetic baroreflexes, through which heart rate and sympathetic vasoconstrictor drive are modulated, respectively. We have previously demonstrated a diminished cardiac baroreflex response to changes in blood pressure in the morning compared with the afternoon (Taylor et al., 2011). It is currently not known whether diurnal variation exists in vascular sympathetic baroreflex function, and thus whether diminished blood pressure control in the morning may also be attributed to poor control of sympathetic outflow to the peripheral vasculature. The hypothalamic paraventricular nucleus (PVN), which directly innervates sympathetic preganglionic neurones in the intermedioloateral cell column of the spinal cord, as well as supplying the rostral ventrolateral medulla (RVLM), receives input from the master body clock (suprachiasmatic nuclei), NTS and RVLM (Blair et al., 1996). The RVLM is the primary output nucleus for sympathetic vasoconstrictor drive (Dampney et al., 2003; Macefield and Henderson, 2010; James et al., 2013) and this pathway may therefore provide a means for the body clock to influence the modulation of muscle sympathetic nerve activity (MSNA) and vascular sympathetic baroreflex function, although no evidence of coupling between PVN and MSNA has been found in humans (James et al., 2013).

The aim of this study is to investigate diurnal variation in vascular sympathetic baroreflex sensitivity (BRS) in young healthy adults. When examining cardiac BRS we have previously employed the modified Oxford method, which is a pharmacological method for assessing baroreflex function (Taylor et al., 2011, 2013). Whilst it is considered the gold standard technique for assessing cardiac BRS (Diaz and Taylor, 2006; Dutoit et al., 2010; Taylor et al., 2014), this pharmacological approach has limitations when used for the vascular sympathetic baroreflex, particularly with regards to increases in arterial pressure following the bolus injection of phenylephrine - when MSNA bursts can be almost entirely inhibited (Dutoit et al., 2010). For the current research question, the baroreflex response to rising pressures is important, given the heightened risk of cardiovascular events linked with acute increases in blood pressure in the morning (Muller et al., 1989). Spontaneous techniques allow baroreflex responses to both rising and falling pressures to be incorporated under resting, physiological conditions. Therefore, in the current study spontaneous methods of assessing vascular sympathetic BRS, previously described by Kienbaum et al. (2001), will be used. It is hypothesized that vascular sympathetic BRS is lower in the morning than in the afternoon, such that increases in blood pressure are subject to less damping.

## Methods

### Participants

The study was conducted with the approval of the Human Research Ethics committee, University of Western Sydney, and satisfied the Declaration of Helsinki. Based on the information presented by Keller et al. (2006), a meaningful difference in vascular sympathetic BRS of −2.0 bursts/mmHg was identified. Previous pilot work performed by our group has provided a standard deviation of differences of 1.6 bursts/100heartbeats/mmHg in vascular sympathetic BRS. From this, it is estimated that a sample size of eight participants will have >80% power to detect a meaningful difference in vascular sympathetic BRS of 2.0 bursts/mmHg, using a paired *t*-test with a 0.05 two-sided significance level. In order to account for unsuccessful experiments and insignificant baroreflex slopes, 12 healthy participants, aged between 19 and 27 years, were recruited. Exclusion criteria included diagnosed cardiovascular, respiratory or endocrine disease, hypertension (>140 mmHg systolic and/or >90 mmHg diastolic blood pressure) and those who smoked or took regular medication. Participants were instructed to abstain from alcohol or vigorous exercise 24 h prior and to not consume any caffeine on the day of both morning and afternoon experiments. Diet was otherwise uncontrolled; subjects studied in the morning had eaten their normal breakfast and those in the afternoon their typical lunch. The changes in hormone levels during the menstrual cycle have been shown to affect MSNA and vascular sympathetic BRS (Minson et al., 2000); accordingly, females were tested in the low hormone (early follicular) phase of their menstrual cycle to minimize the effects of sex hormones on BRS. Written informed consent was obtained from all participants prior to conducting the experiment, who were reminded that they could withdraw at any time.

### Measurements

Participants were studied in an upright-seated position in a comfortable chair, with the legs supported in the extended position. Continuous MSNA recordings were made from muscle fascicles of the common peroneal nerve through tungsten microelectrodes (FHC, Bowdoin, ME, USA) inserted percutaneously at the level of the fibular head. Multi-unit neural activity was amplified (gain 20,000, bandpass 0.3–5.0 kHz) using an isolated amplifier (Neuroamp EX, ADInstruments, Sydney, Australia) and stored on computer (10-kHz sampling) using a computer-based data acquisition and analysis system (Powerlab 16SP hardware and LabChart 7 software; ADInstruments, Sydney, Australia). A root-mean-square (RMS) processed version of this signal was computed, with a moving average of 200 ms. Blood pressure was recorded non-invasively via a finger cuff (Finometer; Finapres Medical System, Amsterdam, the Netherlands). Heart rate was recorded via electrocardiogram (0.3–1.0 kHz, Ag-AgCl surface electrodes, sampled at 2 kHz). Respiration was measured via a strain-gauge transducer (Pneumotrace, UFI, Morro Bay CA, USA) wrapped around the chest.

### Experimental protocol

Participants completed two trials, one beginning at 0800 h and one at 1300 h on two separate days. This was to ensure that, based on time to set up and obtain high quality nerve recordings (approximately 60–90 min), data collection coincided with the times of day associated with high (0900–1000 h) and low occurrence (1400–1500 h) of cardiovascular events during daylight hours (Muller et al., 1989). The order of the two trials was randomized. A minimum of 10 min of resting data was recorded in order to record spontaneous fluctuations in blood pressure and the corresponding changes in MSNA. Participants were not instructed about their breathing.

### Data analysis

Beat-to-beat values were extracted from LabChart (ADInstruments, Sydney, Australia) for systolic pressure, diastolic pressure, R-R interval, and MSNA. A custom-written LabView program was used to detect and measure the area of individual bursts of MSNA. The numbers of bursts per minute (MSNA burst frequency) and per 100 heartbeats (MSNA burst incidence) were determined for each individual.

### Vascular sympathetic baroreflex sensitivity: Burst incidence method

Vascular sympathetic BRS was quantified using methods previously described by Kienbaum et al. (2001). For all methods of assessing vascular sympathetic BRS, the nerve trace was shifted to account for the delay in conduction, and this was adjusted for each participant to account for inter-individual differences in sympathetic burst latency. The average shift applied was 1.24 ± 0.02 s. For each participant, the diastolic pressure values for each cardiac cycle throughout the 10-min rest period were assigned to 3 mmHg bins, removing potential non-baroreflex stimuli (Ebert and Cowley, 1992; Tzeng et al., 2009). For each bin the corresponding MSNA burst incidence (number of bursts per 100 cardiac cycles) was determined. Vascular sympathetic BRS was quantified by plotting MSNA burst incidence against the mean diastolic blood pressure for each bin. Each data point was weighted according to the number of cardiac cycles because the bins at the highest and lowest diastolic pressures contain fewer cardiac cycles (Kienbaum et al., 2001). The value of the slope, determined via linear regression analysis, provided the vascular sympathetic BRS for the individual, which will be referred to as “vascular sympathetic BRS_inc_” in order to differentiate it from other methods of determining vascular sympathetic BRS.

### Vascular sympathetic baroreflex sensitivity: Total MSNA method

The largest MSNA burst during the 10-min rest period was assigned a value of 1000 and the remaining MSNA bursts were calibrated against this to allow measures of MSNA to be normalized to individual resting values (Halliwill, 2000). The relationship between diastolic blood pressure and total MSNA was assessed using 3 mmHg bins. Total integrated MSNA was determined for each bin using a segregated signal averaging approach described by Halliwill (2000) and expressed as arbitrary units (AU) per beat. Linear regression was used to determine the relationship between total MSNA and diastolic blood pressure with the application of the weighting procedure described above to account for the number of cardiac cycles per bin. If threshold or saturation regions were identified, i.e., the presence of 3 or more pressure bins across which there was a plateau in MSNA, then these bins were removed leaving the linear portion of the slope. These baroreflex values will be referred to as “vascular sympathetic BRS_total_” in order to differentiate them from the MSNA burst incidence method for assessing vascular sympathetic BRS.

### Cardiac baroreflex sensitivity: Sequence method

Cardiac BRS was assessed using the sequence method, in which “up” and “down” sequences are identified. “Up” sequences consisted of three or more consecutive cardiac cycles for which there is a sequential rise in both systolic blood pressure and R-R interval. “Down” sequences consisted of three of more cardiac cycles for which there is a sequential fall in systolic blood pressure and R-R interval (Parati et al., 1988). Baroreflex sensitivity was quantified by plotting R-R interval against systolic blood pressure for each sequence (*r* ≥ 0.8 acceptance level) and taking the average slope value for “up” sequences (cardiac BRS_up_), “down” sequences (cardiac BRS_down_), and all sequences pooled (cardiac BRS_pooled_). Values of cardiac BRS were accepted when the number of sequences was ≥3 for both up and down sequences.

### Statistical analysis

Vascular sympathetic BRS values were compared between morning and afternoon using Student's *t*-tests for paired data. All statistical analyses were performed using Prism v6.00 for Mac OS X (GraphPad software, San Diego, California, USA). For all statistical tests, a probability level of *P* < 0.05 (two-tailed) was regarded as significant. All values are expressed as means and standard error (SE).

## Results

### Participants

Twelve participants were recruited for the study. One participant, who reported a family history of hypertension, had a resting blood pressure of 150/82 mmHg and was therefore excluded from the study. Nerve recordings were successfully obtained in all experiments except one afternoon experiment; this participant was excluded from the analysis. Baroreflex sensitivity was therefore assessed on two occasions for 10 participants. Significant vascular sympathetic BRS_inc_, vascular sympathetic BRS_total_, and cardiac BRS values for both the morning and afternoon were acquired for all 10 participants. The mean age of these young, healthy participants was 22 ± 1 year and mean body mass index (BMI) was 23.8 ± 1.0 kg/m^2^.

### Resting cardiovascular variables

**Figure 1:**
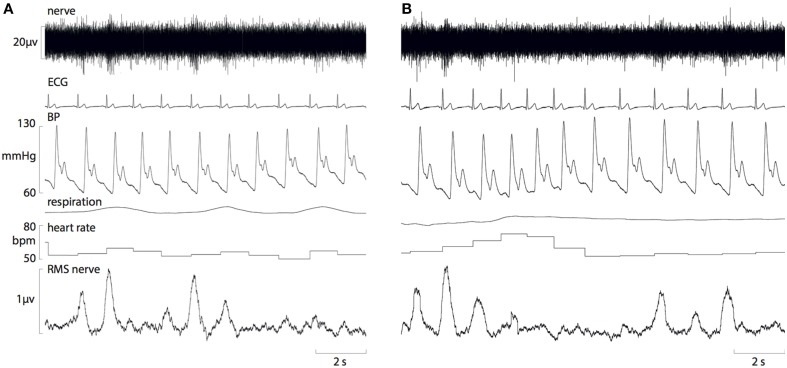
**Raw data recordings of MSNA, ECG, blood pressure and respiration in a 25-year old male in the morning (A) and afternoon (B)**. A fall in diastolic pressure is associated with a baroreflex-driven increase in MSNA, and a rise in diastolic pressure causes inhibition of MSNA bursts.

Resting cardiovascular variables for the 10 participants at both times of day are presented in Table 1. Resting systolic pressure was significantly higher in the afternoon (129 ± 2 mmHg) compared with the morning (120 ± 3 mmHg; *P* = 0.02). However, there were no significant differences in resting diastolic pressure, heart rate or MSNA between the morning and afternoon (*P* > 0.05). Figure 1 shows raw data recordings from one individual in the morning and afternoon.

**Table 1:** **Resting cardiovascular variables in the morning and afternoon (***n*** = 10)**.

**Variable**	**Morning**	**Afternoon**	**Mean difference**	***P***
Systolic pressure (mmHg)	120 ± 3	129 ± 2	9 ± 3^*^	0.02
Diastolic pressure (mmHg)	69 ± 2	70 ± 3	1 ± 2	0.62
Mean arterial pressure (mmHg)	86 ± 2	90 ± 2	3 ± 2	0.11
Heart rate (beats/min)	66 ± 3	68 ± 2	2 ± 2	0.52
MSNA burst frequency (bursts/min)	38 ± 4	37 ± 2	−1 ± 3	0.73
MSNA burst incidence (bursts/100heartbeats)	57 ± 5	58 ± 3	1 ± 5	0.82

*MSNA, muscle sympathetic nerve activity*.

* *Significant difference between morning and afternoon (p < 0.05)*.

### Vascular sympathetic baroreflex sensitivity

**Figure 2:**
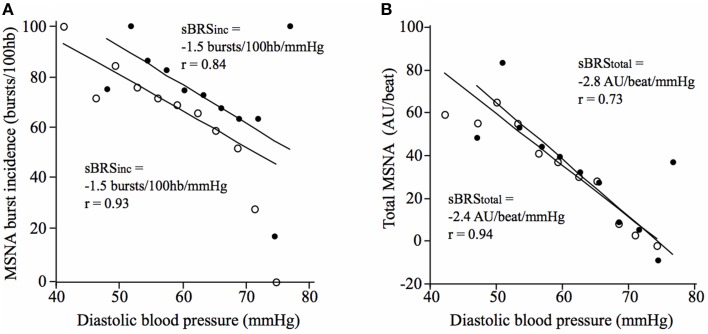
**Vascular sympathetic baroreflex slopes for a 21-year old male in the morning (closed circles) and afternoon (open circles) using (A) the vascular sympathetic BRS_inc_ method, and (B) the vascular sympathetic BRS_total_ method**.

There was no significant difference in vascular sympathetic BRS_inc_ between the morning and afternoon sessions (*P* = 0.68). Similarly, there was no significant difference in vascular sympathetic BRS_total_ between the morning and afternoon (*P* = 0.89). These results are summarized in Table 2. Figure 2 illustrates vascular sympathetic baroreflex slopes in one individual, studied in the morning and in the afternoon on separate days.

**Table 2:** **Vascular sympathetic and cardiac baroreflex sensitivities in the morning and afternoon (***n*** = 10)**.

**Baroreflex sensitivity**	**Morning**	**Afternoon**	**Mean difference**	***P***
Vascular sympathetic BRS_inc_ (bursts/100heartbeats/mmHg)	−2.2±0.6	−2.5±0.2	0.2 ± 0.6	0.68
Vascular sympathetic BRS_total_(AU/beat/mmHg)	−3.0±0.5	−2.9±0.4	0.1 ± 0.6	0.89
Cardiac BRS_pooled_(ms/mmHg)	15.2 ± 1.6	12.5 ± 1.6	−2.7±2.2	0.26
Cardiac BRS_up_(ms/mmHg)	15.3 ± 1.4	12.0 ± 1.6	−3.2±1.9	0.12
Cardiac BRS_down_(ms/mmHg)	15.9 ± 2.3	12.6 ± 1.8	−3.3±2.9	0.29

*BRS, baroreflex sensitivity; AU, arbitrary units*.

### Cardiac baroreflex sensitivity

There was no significant difference in cardiac BRS_pooled_, cardiac BRS_up_, or cardiac BRS_down_ between morning and afternoon sessions (*P* > 0.05; Table 2). Moreover, there was no significant difference between morning and afternoon sessions in the number of cardiac BRS sequences for pooled (31 ± 5 vs. 42 ± 7; *P* = 0.24), “up” sequences (16 ± 3 vs. 20 ± 4; *P* = 0.31) or “down” sequences (16 ± 3 vs. 22 ± 4; *P* = 0.23).

## Discussion

In this study diurnal variation in vascular sympathetic baroreflex sensitivity has been examined for the first time. Previous research indicates that there are two sites for modulation of MSNA: one responsible for burst incidence and the other burst amplitude (Kienbaum et al., 2001). We report that baroreflex modulation of MSNA burst incidence and total MSNA is not significantly different between the morning and afternoon, suggesting that neither site exhibits diurnal variation in the modulation of MSNA.

### Baroreflex sensitivity and time of day

We have previously demonstrated reduced cardiac BRS in the morning compared with the afternoon (Taylor et al., 2011), as assessed using the modified Oxford method. The cardiac and vascular sympathetic baroreflexes share a common afferent arm and therefore we predicted similar diurnal variation in vascular sympathetic BRS. However, no significant difference in vascular sympathetic BRS was observed between the morning and afternoon sessions. Different approaches for assessing the baroreflex were employed for the two studies, which may explain these differences. In our previous work on the cardiac baroreflex we have used the modified Oxford method, which is considered the gold standard technique for studying this arm of the baroreflex (Diaz and Taylor, 2006; Dutoit et al., 2010; Taylor et al., 2014). As previously discussed, this pharmacological approach has potential limitations when used for the vascular sympathetic baroreflex, particularly with regards to increases in arterial pressure (Taylor et al., 2014). Spontaneous techniques allow baroreflex responses to both rising and falling pressures to be incorporated but do not provide the rapid changes in pressure associated with techniques, such as the modified Oxford method, in which blood pressure is actively perturbed (Diaz and Taylor, 2006). Kienbaum and Peters (2004) showed that vascular sympathetic BRS at rest differs from vascular sympathetic BRS quantified during pharmacologically-driven hypotension. It is possible that the baroreflex requires testing under greater and more rapid changes in pressure to reveal significant effects of time of day.

The use of spontaneous techniques may explain why we revealed no significant differences for “up,” “down” or pooled cardiac baroreflex sequences between the morning and afternoon. There was also no significant difference in the number of sequences that occurred at the two times of day. The sequence method is arguably one of the most commonly used spontaneous methods for assessing cardiac baroreflex function. Using this method, Parati et al. (1988) also reported no significant differences in cardiac BRS or number of sequences between the morning (0900–1100 h) and afternoon (1600–1800 h), despite significantly higher values at night (2300–0300 h). Similar findings have been reported in hypertensive patients (Tochikubo et al., 1997). Hossmann et al. (1980) used infusions of noradrenaline to assess cardiac BRS over 24 h. Although this is a pharmacological approach, it is argued that the baroreflex challenge provided by noradrenaline infusions (as opposed to bolus injections) is too gradual, allowing the baroreflex to respond with sufficient changes in heart rate to maintain steady state blood pressure and therefore prevent useful baroreflex slopes from being attained (Diaz and Taylor, 2006; Taylor et al., 2014). Noradrenaline infusions do not provide the rapid changes in blood pressure associated with the bolus injections of sodium nitroprusside and phenylephrine used in the modified Oxford method. Interestingly, the study by Hossmann et al. (1980) revealed significantly higher cardiac BRS at 0300 and 1200 h, and significantly lower values at 0900 and 1500 h. This is consistent with the studies involving spontaneous techniques in which cardiac BRS was high at night and low in the morning and afternoon (Parati et al., 1988; Tochikubo et al., 1997). Our study suggests that both cardiac and vascular sympathetic baroreflex sensitivities, measured under physiological conditions at rest, are not significantly different between the morning and afternoon.

A negative correlation between cardiac BRS and blood pressure responses to stressors has previously been reported (Lipman et al., 2002), suggesting that low BRS is associated with a poor capacity for buffering stress-induced increases in blood pressure. Should diurnal variation in baroreflex modulation of MSNA exist we might expect this to be reflected in the magnitude of the blood pressure response to the cold pressor test, a classic sympathoexcitatory manoeuver. However, we previously demonstrated no significant differences in systolic or diastolic pressure responses to a cold pressor test in the morning and afternoon (Dunn and Taylor, 2014), which is consistent with the lack of diurnal variation in vascular sympathetic BRS in the current study. To our knowledge, no other studies have been performed to assess diurnal variation in vascular sympathetic baroreflex function. However, Nakazato et al. (1998) studied nocturnal variation in vascular sympathetic BRS using a spontaneous approach similar to the sequence method. The method involved identifying sequences of three or more cardiac cycles in which there were sequential increases or decreases in diastolic pressure. Only sequences associated with a negative correlation (regression coefficient < 0) between diastolic pressure and total MSNA were accepted and then entered into a linear regression model to determine the overall baroreflex slope. The authors reported that vascular sympathetic BRS is high at 2300 h but declines during nocturnal sleep, remaining low in the morning (0700 h). However, due to the focus on nocturnal variation, it is not clear from this study when a daytime rise might occur that leads to high vascular sympathetic BRS in the evening. Furthermore, it was reported that microelectrodes had to be re-inserted at least once per participant during the night and it is not clear if this was taken into account by normalizing the MSNA values to the new recording site. Future studies of diurnal variation in vascular sympathetic BRS could incorporate active perturbations in blood pressure, although issues of quantifying responses to rapid rising pressures would need to be considered.

### Resting MSNA

The present study indicates that resting MSNA, when expressed as both burst incidence and burst frequency, does not differ between morning and afternoon. It has been proposed that the morning represents a transition period from low to high sympathetic activity (Panza et al., 1991; Somers et al., 1993; Scheer et al., 2010). It has been shown that MSNA is lower during sleep than wakefulness, except during REM sleep when it exceeds that of wakefulness (Hornyak et al., 1991; Somers et al., 1993). However, no direct comparisons were made between specific times of day so this does not offer insight into variation within daylight hours. In an earlier study, Linsell et al. (1985) reported that whilst noradrenaline exhibits a circadian rhythm this is driven by posture and sleep, with greater levels when individuals are upright and awake. Therefore, we may not expect to observe large differences between the morning and afternoon, but predominantly between periods of sleep and wakefulness. Scheer et al. (2010) later demonstrated circadian variation in sympathetic outflow with a peak in plasma noradrenaline at 0900 h. However, measurements of plasma noradrenaline cannot offer the rapid time resolution that can be achieved with microneurography, which provides a direct measure of sympathetic outflow (Vallbo et al., 2004). Middlekauff and Sontz (1994) used microneurography to measure MSNA in the morning (0630–0830 h) and afternoon (1400–1600 h) and reported no significant effect of time of day on MSNA at rest or in response to lower body negative pressure or handgrip exercise. While the current findings support this previous research, we have further shown that baroreflex modulation of MSNA at rest does not differ between the morning and afternoon.

Finally, Panza et al. (1991) found that forearm vascular resistance was higher and blood flow lower in the morning compared with the afternoon and evening, and that infusions of phentolamine (α-adrenergic antagonist) eliminated the time-of-day differences in vascular resistance. The authors therefore concluded that greater sympathetic vasoconstriction in the morning is responsible for the elevated vascular resistance. This may suggest that, whilst MSNA has been shown to be consistent between morning and afternoon, the end-organ response may be greater in the morning. To date there have been no studies of diurnal variation in neurovascular transduction. Greater vascular transduction of MSNA in the morning may explain elevated vascular resistance and contribute to the higher incidence of cardiovascular events at this time of day, though we cannot provide any mechanistic insight into how this augmented vascular transduction comes about.

### Limitations

The findings of the current study are limited to healthy young populations and may not be extrapolated to older and/or hypertensive populations. Future research is required to assess diurnal variation in vascular sympathetic BRS in aging populations and those at risk of cardiovascular events. Although the higher resting systolic pressure in the afternoon was surprising, importantly resting diastolic pressure was not significantly different between the two times of day. It is the changes in diastolic pressure that drive MSNA, and thus diastolic pressure is more closely correlated with MSNA (Sundlöf and Wallin, 1978). The current findings indicate that diastolic pressure, resting MSNA burst incidence and the relationship between the two are consistent between the morning and afternoon. This is in contrast to our previous findings in cardiac BRS, in which the morning was associated with diminished cardiac baroreflex function (Taylor et al., 2011). This may be explained by the use of the modified Oxford method in the previous study; the current findings may be limited by the use of spontaneous techniques for assessing vascular sympathetic BRS.

## Conclusion

In this study diurnal variation in vascular sympathetic baroreflex sensitivity was examined for the first time. The findings indicate that baroreflex modulation of MSNA burst incidence and total MSNA does not differ between morning and afternoon at rest. Future research using methods to actively perturb blood pressure would allow diurnal variation in vascular sympathetic baroreflex control during rapid changes in pressure to be explored. Further research is required to determine whether vascular transduction of MSNA differs with time of day.

## Author contributions

Experiments were performed in the School of Medicine (University of Western Sydney). All authors were involved in the design of the experiments and/or acquisition and analysis of the data, as well as the writing or editing of this manuscript. All authors approved the final version of the manuscript and agree to be accountable for all aspects of the work.

### Conflict of interest statement

No authors (or their institutions) of this manuscript received payment or services from a third party for any aspect of this manuscript. We have no financial relationships with entities perceived to influence the research presented in this manuscript, nor do we have patents or copyrights to declare. We are unaware of any other potential relationships or activities that might have influenced the writing of this manuscript.
